# Calculation of Myocardial Percentage Collagen, Mast Cell Count and Presence of Arteriosclerosis in Dogs With Myxomatous Mitral Valve Disease and Control Dogs–A Pilot Study

**DOI:** 10.1002/vms3.70689

**Published:** 2025-11-17

**Authors:** Annie Harlow, Matthew Leong, Eliza Talbot‐Williams, Sophie McGill, Alexander Stoll, Ross Harley, Melanie Hezzell

**Affiliations:** ^1^ Bristol Veterinary School University of Bristol Langford House Langford Bristol UK; ^2^ Medivet Roehampton London UK; ^3^ Roundwood Vets London UK

**Keywords:** arteriosclerosis, canine, collagen, dog, myxomatous mitral valve disease

## Abstract

**Objectives:**

This study aimed to (1) determine the feasibility of calculating percentage myocardial collagen (PMC) in formalin‐fixed, paraffin‐embedded canine tissue samples, (2) explore relationships between PMC, mast cell (MC) count and myocardial arteriosclerosis/arteriolosclerosis (MA) and (3) calculate the sample size required to compare PMC between dogs with and without myxomatous mitral valve disease (MMVD).

**Methods:**

Histological sections were prepared from retrospective samples of formalin‐fixed, wax‐embedded ventricular myocardial tissue from 15 dogs with, and seven dogs without, MMVD. Sections from each sample were stained with Masson's trichrome, haematoxylin and eosin (H&E) or toluidine blue. In each Masson's trichrome‐stained section, digital images of 12 random fields were captured and the total image area and total collagen area were measured using computer‐assisted morphometric analysis. Mean collagen area, mean total image area and mean PMC ([mean collagen area/mean total area] × 100) were calculated per sample. MCs were counted manually in toluidine blue‐stained sections and the mean MC count was calculated from 10 fields. MA was identified by visual inspection of transversely cut vessels in each H&E‐stained section.

**Results:**

No significant difference was detected between MMVD and control dogs in PMC (*p *= 0.063), MC (*p *= 0.476) or MA (*p *= 0.172). A sample size calculation suggests that data from at least 26 MMVD dogs and 26 controls are required to detect a difference in PMC. No significant association was detected between PMC and MC count (*p *= 0.606) in cases of MMVD.

**Clinical Significance:**

The methods described are feasible and the sample size for a definitive study has been estimated.

## Graphical abstract

This pilot study explored the feasibility of calculating myocardial percentage collagen (PMC), mast cell (MC) count and assessing arteriosclerosis in canine myocardial tissue. No significant difference in PMC, MC count or arteriosclerosis was detected between dogs with and without myxomatous mitral valve disease (MMVD). A sample size calculation suggests that at least 26 dogs per group are needed for future studies to determine whether a true difference in myocardial fibrosis exists.

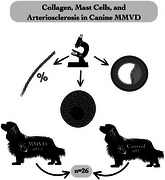

## 1 Introduction

Myxomatous mitral valve disease (MMVD) in dogs results in eccentric hypertrophy of the left ventricle in response to the development of mitral regurgitation (Ljungvall et al. 2011). This process involves breakdown of collagen in the extracellular matrix, which is thought to be necessary before the ventricle can dilate (Stewart et al. 2003). However, myocardial fibrosis, in which new collagen is laid down, also develops with the progression of MMVD (Falk et al. 2013). Thus, the relationship between collagen production and breakdown as MMVD progresses is complex (Hezzell et al. 2014).

Previous studies in dogs have assessed the severity of myocardial fibrosis using semi‐quantitative methodologies, such as severity scoring, rather than quantitative methods like calculation of percentage myocardial fibrosis, which has been reported in human patients (Klappacher et al. 1995). Therefore, there is a need to assess whether similar quantitative methods can be applied in canine veterinary patients.

Mast cells release both profibrotic mediators (e.g., cytokines and chemokines) and enzymes that degrade collagen (e.g., matrix metalloproteinases; Stewart et al. 2003). Increased mast cell density has been demonstrated at sites of fibrosis in the endomyocardium in human patients (Li et al. 1992) and in the mid‐ventricular myocardium in canine experimental models of acute mitral valve regurgitation. It is therefore plausible that mast cells might play a role in mediating myocardial collagen deposition and degradation in canine MMVD.

MMVD in dogs is also associated with arteriosclerosis (Falk et al. 2010). The arteriosclerosis is believed to compromise myocardial blood supply, leading to ischaemic injury and promotion of fibrosis (Nikolic et al. 1988; Falk et al. 2013). It has been hypothesised that the formation of fibrotic tissue within the myocardium amplifies hypoxia by reducing the vasodilatory reserve, leading to further ischaemia and cell death (Falk et al. 2013). An increase in either myocardial arteriosclerosis or fibrosis has been associated with decreased survival times in dogs with naturally occurring congestive heart failure (Falk et al. 2010).

The aims of this pilot study were to (1) determine the feasibility of calculating percentage myocardial collagen in formalin‐fixed, paraffin embedded tissue samples obtained from dogs with naturally occurring heart disease, (2) explore relationships between percentage myocardial collagen, mast cell count and myocardial arteriosclerosis and (3) obtain data for calculation of the sample size required to compare percentage myocardial collagen between dogs with MMVD and control dogs without MMVD to inform the design of a larger, prospective study.

## 2 Materials and Methods

### 2.1 Study Design and Case Selection Criteria

Ethical approval was granted by (University of Bristol Animal Welfare and Ethical Review Body) (VIN/18/047).

The post‐mortem reports and submission forms from dogs submitted to the (University of Bristol Veterinary School tissue bank) post‐mortem service from 2014–2019 were reviewed to identify control and MMVD cases with archived formalin‐fixed, wax‐embedded myocardium samples and for which consent for research use was indicated or had not been withdrawn. Control cases were required to be a minimum of 4 years of age with no cardiac disease or other disease process affecting the heart indicated in the clinical history or post‐mortem report. MMVD cases comprised dogs of any age and breed with MMVD confirmed on post‐mortem examination by the attending pathologist and without any other cardiac diseases, including neoplastic disease affecting the heart, identified in the clinical history or post‐mortem report. Any dog in either group with evidence of systemic disease or disease other than MMDV that could be associated with fibrosis was excluded. Additionally, following preparation of histology sections, any case with evidence of severe histological freeze‐thaw artefacts affecting the myocardium (e.g., from cold storage of the cadaver prior to the post‐mortem examination) was excluded. In cases where more than one myocardial sample had been processed by the attending pathologist, each parameter was evaluated in one slide per sample and the average measurement per case was reported. The location of sample acquisition and the number of samples processed were not standardised, but all samples were taken from the left ventricular free wall or interventricular septum.

The samples had been fixed in neutral buffered formalin before being processed for embedding in paraffin and stored as paraffin blocks.

Histological examination of each myocardial sample was performed on three sections, 4 µm thick, and stained with Masson's trichrome, haematoxylin and eosin (H&E), or toluidine blue.

### 2.2 Calculation of Percentage Myocardial Collagen

Calculations of percentage myocardial collagen were performed by one of the authors (Annabelle Harlow). Using a Leica DMR light microscope fitted with a Leica DC350FX camera and connected to Photoshop 5 (Adobe, San Jose, California, USA), images at 12 random fields (×20 objective) on each Masson's trichrome‐stained slide were captured. At each field, three monochromatic images (red, green and blue) were captured by the camera and subsequently combined in Photoshop to create a single colour image for subsequent analysis.

**FIGURE 1:**
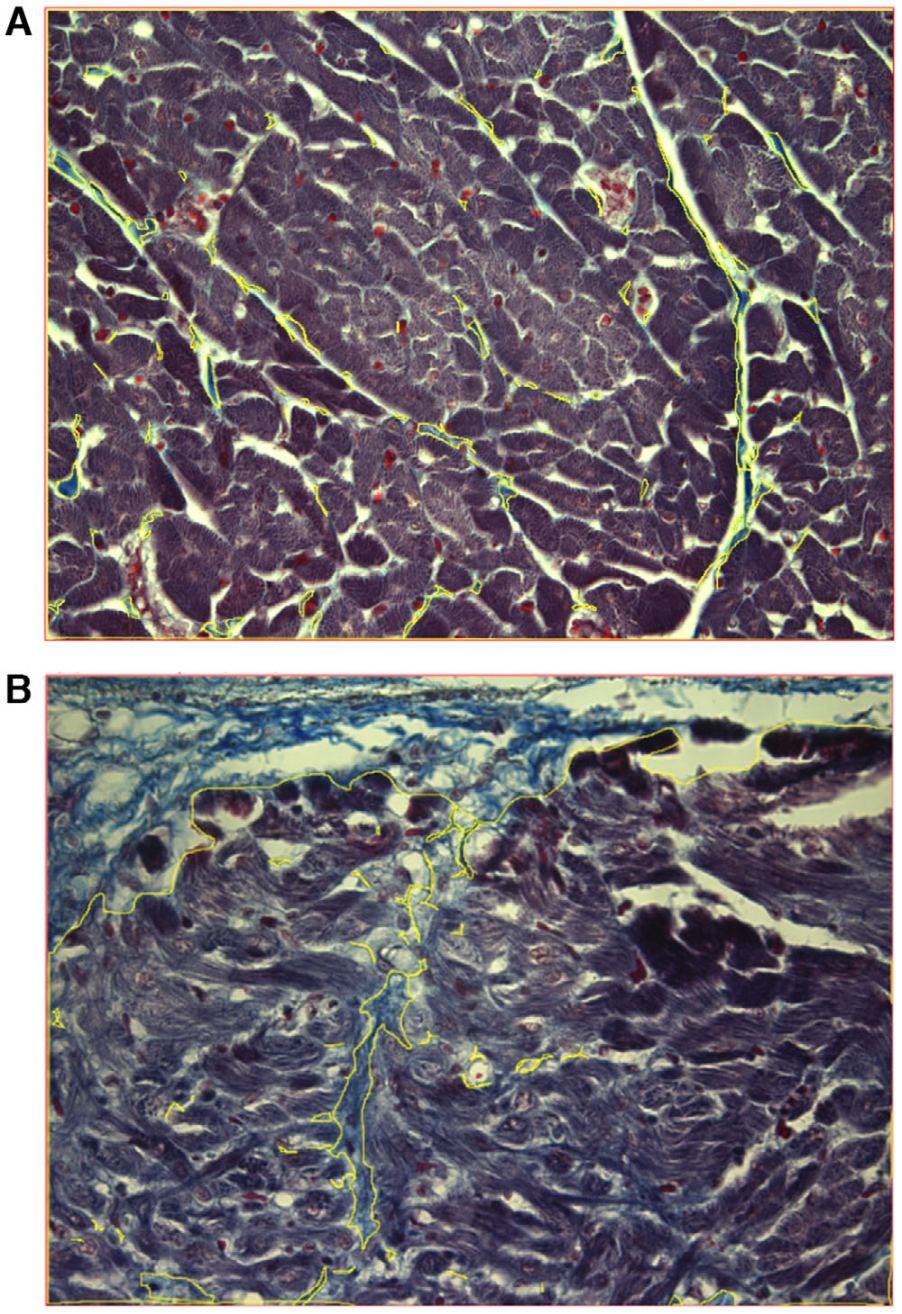
(A) Two representative images (×20 objective) captured from sections of Masson's trichrome stained canine ventricular myocardium from two different cases (both MMVD cases). Blue‐stained tissue (collagen) is outlined in yellow, and the total image area is outlined in red using image analysis software (ImageJ) to demonstrate the method of segmenting the area used to calculate myocardial percentage collagen ([collagen area/total image area] × 100). The area of collagen in the top left‐hand corner of (B) represents an example which includes subendocardial collagen.

Using ImageJ (National Institute of Health, Bethesda, Maryland, USA), the area in pixels occupied by collagen within each image was calculated by outlining all blue‐stained tissue on the slides stained with Masson's trichrome (Figure 1A,B). The total image area, in pixels, was calculated by drawing around the total area of each image (Figure 1A,B). For each section, the results from all 12 images were used to calculate the mean collagen area and mean total area for each sample. The mean percentage myocardial collagen for each case was calculated using the formula (mean collagen area/mean total image area) ×100. For some of the dogs, more than one myocardial sample was available for analysis. In each of these cases, the mean percentage myocardial collagen was calculated for each available myocardial sample, and then these values were averaged to produce an overall mean myocardial percentage collagen value for that dog. In each case, only the overall mean percentage collagen value for the dog is reported.

### 2.3 Assessment of Myocardial Arteriosclerosis/Arteriolosclerosis

**FIGURE 2:**
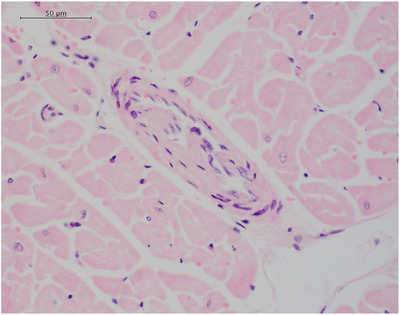
A histologic micrograph demonstrating the presence of atheroma plaques in a section of Masson's trichrome‐stained canine ventricular myocardium.

Evidence of arteriosclerosis/arteriolosclerosis was assessed, and recorded as present or absent, based on the morphological changes described and illustrated by Falk et al. (2006) and Suzuki et al. (1994) (proliferative, hyaline or obstructive changes). In cases where more than one myocardial tissue sample was available for analysis, arteriosclerosis/arteriolosclerosis was recorded as present if changes were present in any of the available samples. The assessment was performed by a single veterinary pathologist (Alexander Stoll) using the H&E‐stained slides (Figure 2). Difficulty in the interpretation of the vascular morphology was associated with the plane of section, whereby tangential sections through vessels limits interpretation. The majority of the blood vessels identified within the tissues were arterioles; therefore, the term arteriolosclerosis will be used for the remainder of the manuscript.

### 2.4 Myocardial Mast Cell Counts

**FIGURE 3:**
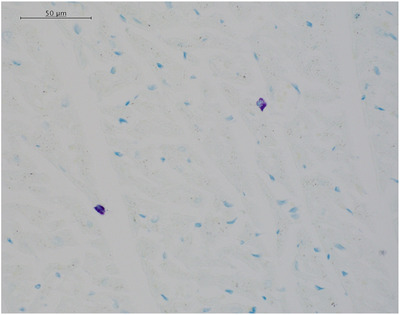
A representative image (×20 objective) showing mast cells within canine ventricular myocardium.

For each sample, mast cells, identified by morphology and metachromatic staining (Figure 3), were counted manually under light microscopy in a toluidine blue‐stained section in 10 randomly‐selected high‐power (× 40 objective) fields using a battlement technique and then averaged. For the dogs in which more than one myocardial sample was available for analysis, the mean mast cell count was calculated for each available myocardial sample and then these values were averaged to produce an overall mean mast cell count for that dog. In each case, only the overall mean mast cell count for the dog is reported. The counts were performed by a single veterinary pathologist (Alexander Stoll) who was blinded to the group (control vs. MMVD).

### 2.5 Statistical Analysis

Statistical analyses were performed using commercially available software (SPSS for Windows version 24.0, SPSS Inc, Chicago, Illinois, USA; and GraphPad Prism version 9.4, GraphPad Software Inc, California, USA). Data were assessed for normality graphically and by use of the Shapiro–Wilk test. Comparisons of continuous variables between groups were performed using Mann–Whitney U tests. Comparisons of proportions between groups were made using chi‐square or Fisher's exact tests, as appropriate. Associations were assessed using simple linear regression. The residuals were assessed for normality by visual assessment of Q–Q plots. A value of *p* < 0.05 was considered significant. The sample size calculation was calculated using the ‘Sample size calculator for two sample Wilcoxon Mann–Whitney U‐test’ available via the University of Vienna (https://homepage.univie.ac.at/robin.ristl/samplesize.php?test=wilcoxon).

## 3 Results

Suitable samples of formalin‐fixed, wax‐embedded ventricular myocardial tissue from 22 cases that met the selection criteria were available. These comprised 15 dogs with MMVD (MMVD cases) and seven dogs without MMVD (control cases). The number of myocardial samples per case ranged from 1 to 4 (median = 2). In all cases, the dogs’ owners had given permission for tissues to be retained and used for research.

The summary statistics for each group are presented in Table 1. Body weight was not recorded for three dogs in the MMVD group. No significant difference in age (*p* = 0.322), sex (*p* = 0.583) or body weight (*p* = 0.142) was detected between groups (Table 1).

**TABLE 1:** Signalment, body weight, frequency of arteriolosclerosis and time between death and post‐mortem examination for the dogs included in the study.

	Control group (*n* = 7)		Myxomatous mitral valve disease group (*n* = 15, unless otherwise stated)		
Variable	Median (minimum, maximum)	Number (%)	Median (minimum, maximum)	Number (%)	*p* value
Age (years)	6.8 (4.6, 9.3)		8.8 (4.0, 12.0)		0.322
Breed					N/A
Bichon Frisé		1 (14.3%)		0	
Border Collie		1 (14.3%)		0	
Cairn Terrier		1 (14.3%)		0	
Cavalier King Charles Spaniel		0		2 (13.3%)	
Crossbreed		1 (14.3%)		5 (33.3%)	
German Shepherd		1 (14.3%)		0	
Jack Russel Terrier		1 (14.3%)		2 (13.3%)	
Labradoodle		0		1 (6.7%)	
Labrador Retriever		0		1 (6.7%)	
Maltese Terrier		0		1 (6.7%)	
Siberian Husky		0		1 (6.7%)	
Springer Spaniel		0		1 (6.7%)	
Staffordshire Bull Terrier		0		1 (6.7%)	
West Highland White Terrier		1 (14.3%)		0	
Sex (male/ female)					0.583
Male (entire)		1 (14.3%)		3 (20.0%)	
Male (neutered)		2 (28.6%)		8 (53.3%)	
Female (entire)		1 (14.3%)		1 (6.7%)	
Female (spayed)		3 (42.9%)		3 (20.0%)	
Body weight (kg)	9.9 (5.3, 30.0)		19.0 (6.5, 26.5) (*n* = 12)		0.142
Arteriosclerosis/ arteriolosclerosis					0.397
Present		4 (57.1%)		5 (33.3%)	
Absent		3 (42.9%)		9 (60.0%)	
Missing data (haematoxylin and eosin slide damaged)		0		1 (6.7%)	
Approximate time between death and post‐mortem examination (h)	21 (12, 72)		13 (2, 96)		

Abbreviation: N/A, not applicable.

**FIGURE 4:**
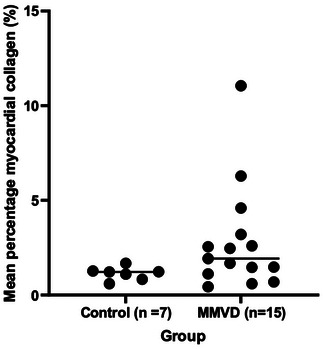
Dot plots of percentage myocardial collagen measurements in 15 dogs with MMVD and seven control dogs. The horizontal central lines represent the median value.

No significant difference in percentage myocardial collagen was detected between the control (median = 1.22% [minimum = 0.60%, maximum = 1.68%]) and MMVD (1.93% [0.4%, 11.1%]) groups (*p* = 0.063; Figure 4).

**FIGURE 5:**
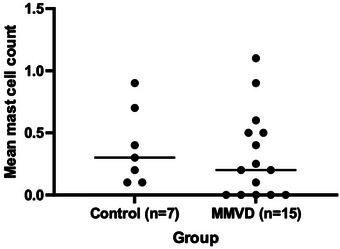
Dot plots of mean mast cell counts in 15 dogs with MMVD and seven control dogs. The horizontal central lines represent the median value.

No significant difference was detected between the mean mast cell count in the control (0.3 [0.1, 0.9]) and MMVD (0.2 [0, 1.1]) groups (*p* = 0.476; Figure 5)

**FIGURE 6:**
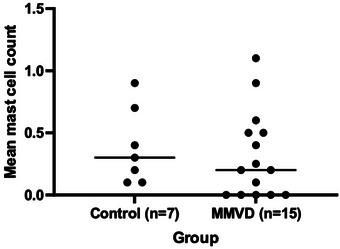
Scatter plot of mean percentage myocardial collagen versus mean mast cell count in the MMVD group. The black points represent individual dogs. The solid line represents the linear aggression line, with the upper and lower 95% confidence intervals for the regression line illustrated by the dashed lines.

No evidence of a significant association between mean mast cell count and mean percentage myocardial collagen was detected in the MMVD group (*p* = 0.606; Figure 6).

No significant difference in the proportion of dogs with evidence of arteriosclerosis was detected between groups (*p *= 0.397; Table 1).

A sample size calculation suggested that 26 dogs with MMVD and 26 control dogs would be required to investigate whether differences in percentage collagen exist between groups (*α *= 0.05, *β* = 0.8, *p*(*X *> *Y*) = 0.274).

## 4 Discussion

This pilot study demonstrated that our technique for calculation of myocardial percentage collagen was feasible in formalin‐fixed, paraffin‐embedded tissue samples obtained from clinical veterinary patients.

Furthermore, these pilot data have facilitated a sample size calculation, which suggests data from a minimum of 26 dogs with MMVD and 26 controls would be required to reliably determine whether a difference in percentage myocardial collagen exists between groups.

No significant association was demonstrated between mean mast cell number and percentage collagen in samples of myocardium from dogs with MMVD. This finding is in contrast to Stewart et al. (2003), who demonstrated a positive relationship between mast cell density and extracellular matrix degradation, contributing to left ventricular eccentric hypertrophy in an experimental canine model of mitral regurgitation. Similar relationships have been demonstrated in rats with the use of pressure overload models (Levick et al. 2009), suggesting that the relationship between mast cells and myocardial remodelling is not model‐ or species‐specific. It is likely that the development of fibrosis involves both pro‐ and anti‐fibrotic stages and is not a linear process. Thus, it is possible that changes in mast cell density may occur before evidence of increased myocardial collagen content develops. Such relationships may be challenging to identify and changes in mast cell density may be highly localised to areas of fibrosis (Olivetti et al. 1989; Li et al. 1992). In the present study, mean myocardial percentage fibrosis and mean mast cell counts were determined separately by two different operators, blinded to one another's findings. As a result, different areas of each slide were inevitably selected for analysis, which may have impacted the ability to demonstrate such a relationship. Alternatively, it is possible that mast cells are not important mediators of ventricular remodelling in dogs with spontaneous MMVD.

The use of Masson's trichrome‐stained slides allowed assessment of the percentage collagen present in the sample sections. However, not all of this connective tissue will represent fibrosis. The images acquired for each section were from randomly chosen locations of ventricular myocardium and some of these images included areas where collagen may be relatively abundant, such as the subendocardium, and in perivascular locations, especially around larger vessels. In the future, any images containing these structures should be assessed visually so that areas that normally have abundant connective tissue can be excluded from the analysis (MacLeod et al. 1994).

In addition to those previously discussed, the present study has a number of limitations. Some are inherent to pilot studies; for example, the study was underpowered to demonstrate differences between groups. Also, the use of samples selected retrospectively from a tissue archive meant that the location of sampling of myocardial tissue could not be standardised. This is an important consideration, as the severity of fibrosis has been shown to differ significantly between different areas of the heart in dogs with MMVD, for example, Falk et al. (2006) demonstrated that in canine MMVD, fibrosis is not distributed consistently throughout the myocardium but is most pronounced subendocardially and in papillary muscles. There was also an absence of grading of MMVD severity amongst the affected dogs, as a result of the retrospective nature of the data. Given that disease grade is likely to influence the degree of myocardial fibrosis, the lack of this information may have contributed to the absence of statistically significant differences observed in collagen percentage. Therefore, in future studies, ideally, tissue samples should be collected prospectively to enable standardisation of the sample location(s) and inclusion of disease grading. Furthermore, as discussed above, future studies should ensure that the same areas of each slide are assessed for parameters such as myocardial percentage collagen and mean mast cell count.

When assessing arteriolosclerosis, it is important that the arterioles are presented in cross‐section (i.e., the arterioles are orientated perpendicular to the section). In many of the sections used in the present study, the vessels were not appropriately orientated, or the size of the tissue section resulted in small numbers of arterioles being present, making the accurate assessment of arteriolosclerosis challenging. Future studies should aim to investigate methods to achieve the most appropriate orientation and sectioning of myocardial tissue samples to enable accurate assessment of arteriolosclerosis.

The potential for post‐mortem changes in the samples analysed also merits consideration. Factors influencing post‐mortem changes in tissues can include the post‐mortem interval and the storage conditions (e.g., ambient temperature) that the cadaver is subject to during this time period. In the post‐mortem reports in which the post‐mortem interval was specified, this varied from 2 to 96 h. Additionally, some cadavers had artefacts relating to autolysis. These factors may have affected the degree of tissue degradation present, which could influence the results. For example, if post‐mortem degradation or freeze‐thawing artefacts affecting various tissue components were to alter their volume and spatial arrangements, this could adversely affect parameters measuring their relative areas. In future studies, samples should be collected prospectively using standardised methods, including limiting the time between death and sample acquisition and avoiding freezing of cadavers prior to post‐mortem examination, with the aim of limiting between‐sample variability.

Finally, although several medications have antifibrotic effects (e.g., angiotensin converting enzyme inhibitors and spironolactone), the medications the patients had received prior to death were not recorded on the post‐mortem reports, therefore, any effects that such drugs may have had on the results are unknown. Future studies of myocardial fibrosis in veterinary patients should consider appropriate methods to gather information on the medications received by each patient.

In conclusion, this study has demonstrated the successful application of a method to calculate myocardial percentage collagen in formalin‐fixed, paraffin‐embedded samples obtained at post‐mortem examination from clinical canine patients. Using this method, we have established that a minimum of 26 MMVD dogs and 26 healthy controls are needed to investigate the hypothesis that the percentage myocardial collagen is higher in dogs with MMVD than in healthy control dogs. Further research is warranted to explore relationships between mean myocardial percentage collagen, mean mast cell counts and severity of arteriosclerosis.

## Author Contributions

**Annie Harlow**: data curation, methodology, writing – original draft, writing – review and editing. **Matthew Leong**: data curation. **Eliza Talbot‐Williams**: data curation. **Sophie McGill**: data curation. **Alexander Stoll**: data curation. **Ross Harley**: supervision, writing – review and editing. **Melanie Hezzell**: conceptualisation, funding acquisition, supervision, writing – review and editing.

## Funding

This study was supported by a grant from BSAVA PetSavers (SRP 01.19).

## Ethics Statement

This study was approved by the appropriate ethics review committee (University of Bristol Animal Welfare and Ethical Review Body) and adheres to international, national, and institutional guidelines for humane animal treatment and complies with relevant legislation, VIN/18/047.

## Conflicts of Interest

The authors declare no conflicts of interest.

## Peer Review

The peer review history for this article is available at https://doi.org/10.1002/vms3.70689.

## Data Availability

The data that support the findings of this study are available from the corresponding author upon reasonable request.
